# Supplementary material for: Collapse of carbon nanotubes due to local high-pressure from van der Waals encapsulation
Source: Nat Commun. 2024 Apr 25;15:3486. doi: 10.1038/s41467-024-47903-3 (PMC11045769; doi:10.1038/s41467-024-47903-3)
Supplement: Supplementary file 1 — Supplementary Information [file 41467_2024_47903_MOESM1_ESM.pdf]

Supplementary Information for

Collapse of carbon nanotubes due to local high-pressure from van der Waals encapsulation

Cheng Hu[1,2,†], Jiajun Chen[1,2, †], Xianliang Zhou[1,2, †], Yufeng Xie[1,2, †], Xinyue Huang[1,2], Zhenhan Wu[1,2], Saiqun Ma[1,2], Zhichun Zhang[1,2], Kunqi Xu[1,2], Neng Wan[3], Yueheng Zhang[1,2], Qi Liang[1,2,4], Zhiwen Shi[1,2,4]*

[1]Key Laboratory of Artificial Structures and Quantum Control (Ministry of Education), School of Physics and Astronomy, Shanghai Jiao Tong University, Shanghai 200240, China.

[2]Collaborative Innovation Center of Advanced Microstructures, Nanjing 210093, China.

[3]Key laboratory of MEMS of Ministry of Education, School of Integrated Circuits, Southeast University, Nanjing 210096, China

[4]Tsung-Dao Lee Institute, Shanghai Jiao Tong University, Shanghai 200240, China.

[†]These authors contributed equally to this work.

*To whom correspondence should be addressed. Email: zwshi@sjtu.edu.cn

Suppl. Note 1. Effect of coverage thickness on CNT height measurement

Suppl. Note 2. Near-field infrared and topography images of the collapsed CNT in Fig. 1b

Suppl. Note 3. More Raman data of collapsed CNTs.

Suppl. Note 4. Estimation of the pressure and strain distribution in collapsed CNT

Suppl. Note 5. Few-layer graphene encapsulated CNT

Suppl. Note 6. Fully atomistic MD simulation of CNT encapsulated by hBN

Suppl. Note 7. Theory of the Levy-Carrier (LC) model

Suppl. Note 8. Luttinger-liquid theory of plasmons in CNTs

Suppl. Note 9 Encapsulated CNTs simulated under different strains in the upper layer

Suppl. Note 10. More examples for the collapse induced metal-semiconductor transition in CNTs

Suppl. Note 11. Near-field optical map of typical distribution of hBN-encapsulated CNTs

Suppl. Note 1. Effect of coverage thickness on CNT height measurement

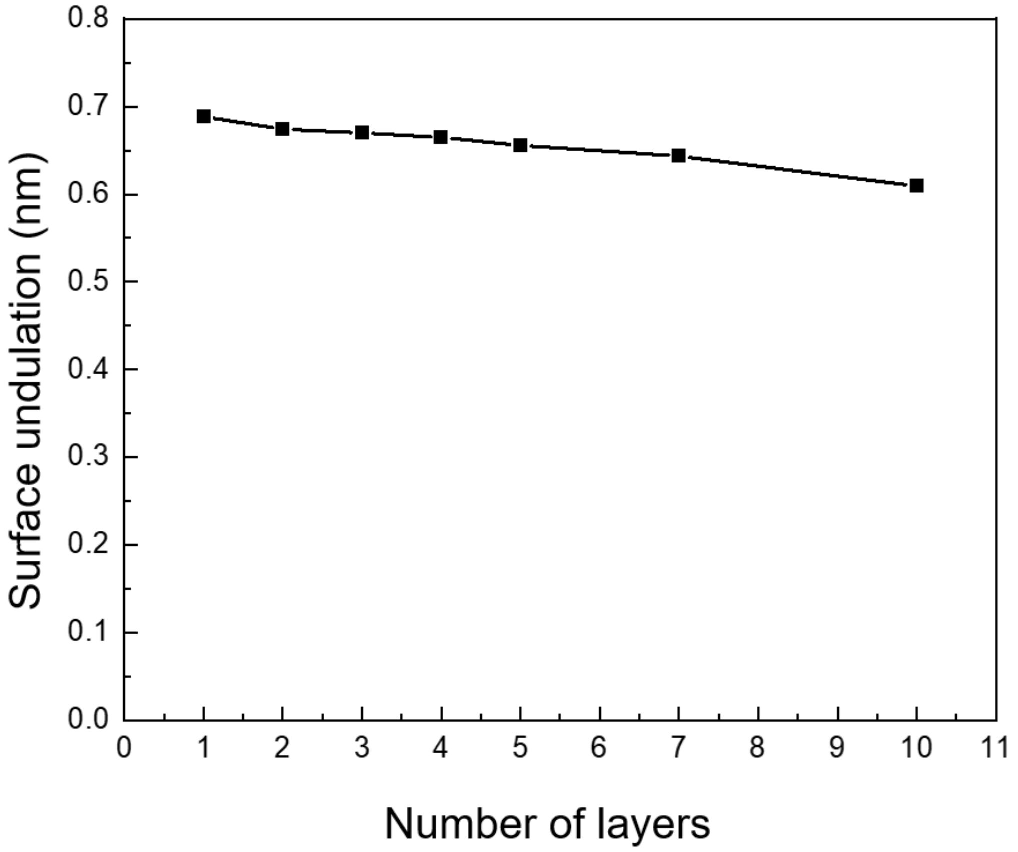

Supplementary Figure 1. The dependence of the surface undulation of the top layer as a function of the number of layers.

Different thicknesses of vdW layers will lead to different heights of encapsulated CNTs measured by AFM. Here we simulate the effect of the thickness of the coverage graphene on the height measurement of the carbon tube. The surface undulation corresponding to a typical 3-nm-thick graphene (≈10 layers) in the experiment is only 0.06 nm, smaller than the surface undulation obtained by the carbon nanotube encapsulated by the tri-layer graphene in the main text.

Suppl. Note 2. Near-field infrared and topography images of the collapsed CNT in Fig. 1b

In Suppl. Fig. 2, we show the CNT encapsulated by hBN with a portion of the CNT collapsed. Suppl. Fig. 2a shows the near-field distribution of the CNT. The bottom part of the CNT is exposed the outside of the hBN, which shows the strongest near-field response, indicating its metallicity. The rest of the CNT is encapsulated underneath the hBN. The near-field response of the CNT encapsulated underneath the hBN is weaker than that of the exposed part, but it still shows a higher near-field intensity than the surrounding hBN response. Interestingly, in the middle part of the CNT become darker in the near-field response. In order to understand what happened to the CNT in between, we imaged its topography in-situ, as shown in Suppl. Fig. 2b. Then, we zoom in to the point at which the near-field change occurs, as shown Suppl. Fig. 2c. The topography of The CNT showed a change in height near the point of abrupt change where the near-field change occurred. In Suppl. Fig. 2d, we can see more clearly that the change in topography and near-field intensity show a high degree of agreement. This phenomenon also provides evidence that the structure transformation of CNTs leads to a metal-semiconductor transition.

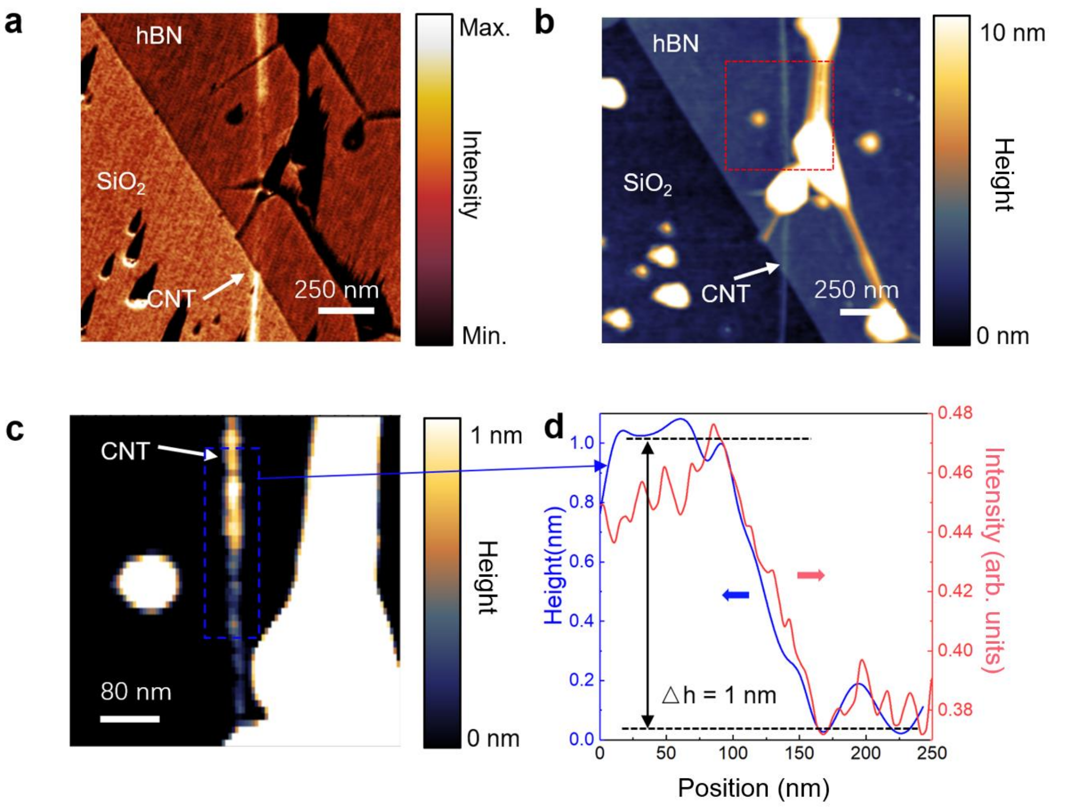

Supplementary Figure 2. Large-area near-field infrared and topography images of the collapsed CNT shown in Fig. 1b. a, the near-field infrared image, and **b,** the topography image of the collapsed CNT underneath hBN encapsulation. **c,** the zoom-in topography of the collapsed CNT in (b) marked by the red dashed rectangle. **d,** correspondence between the topography line-profile and the in-situ near-field infrared line-profile of the partially collapsed CNT.

Suppl. Note 3. More Raman data of collapsed CNTs

More Raman data are shown as Suppl. Fig. 3.

We provide below more Raman spectra taken from a large number of samples, shown in Suppl. Fig. 3a (from collapsed CNTs) and Suppl. Fig. 3b (from free-standing CNTs). These two sets of Raman spectra exhibit obvious differences in the G- and D-peak. First, the G-peak of all the collapsed-CNT Raman spectra becomes much broader than these for the free-standing ones. This is displayed more clearly in Suppl. Fig. 3c. Second, the Raman D-peak grow prominently for the collapsed CNTs. These two differences provide clear evidence for the structural collapse of the CNTs.

Furthermore, we have included Raman mapping (Suppl. Fig. 3 d-g) of a CNT that exhibits collapsed and free-standing structure, clearly demonstrating differences in D-

peak intensity and G-peak width between the collapsed and free-standing parts. Suppl. Fig. 3d is the topography image of both collapse and free-standing configurations, half underneath the hBN encapsulation and half expose in the outside. Suppl. Fig. 3 e-g represent the near-field image, intensity of D peak mapping, FWHM of G peak mapping of (a), respectively.

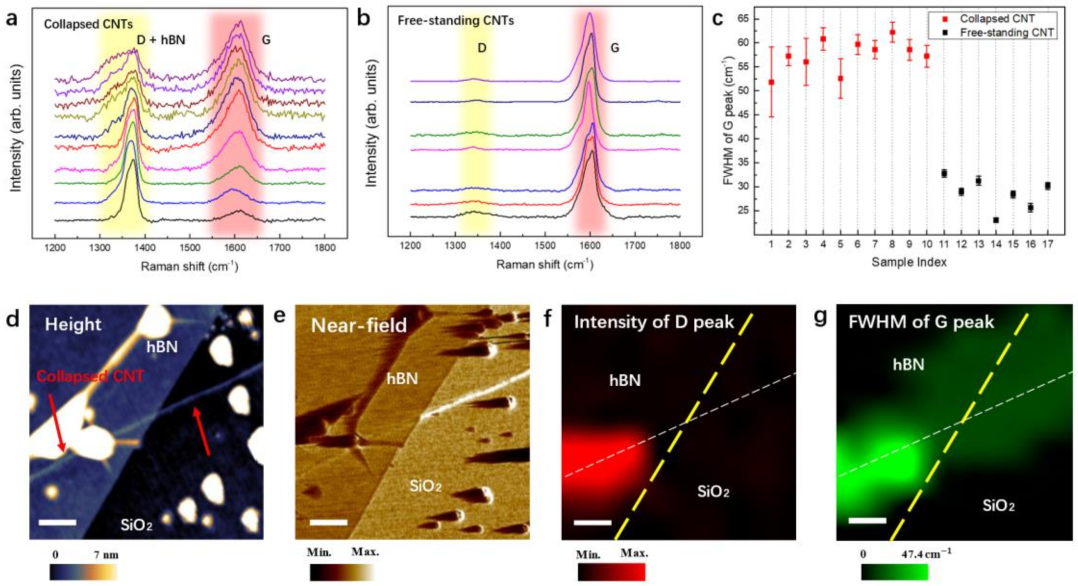

Supplementary Figure 3. More Raman data of collapsed CNTs. a, Raman spectra of more collapsed CNTs. **b**, Raman spectra of more free-standing CNTs. The data in (e) and (f) is collected from different points at different CNTs insides or outsides the hBN-encapsulated area randomly. **c**, the distribution of FWHM of G peak of collapsed CNTs(red) and free-standing CNTs(black) in (a)(b). The error bars represent the standard error of the fitting FWHM. **d**, topography image of both collapse and free-standing configurations, half underneath the hBN encapsulation and half expose in the outside. **e**, **f**, **g**, near-field image, intensity of D peak mapping, FWHM of G peak mapping of (a), respectively. Scale bar: 250 nm

Suppl. Note 4. Estimation of the pressure and strain distribution in collapsed CNT

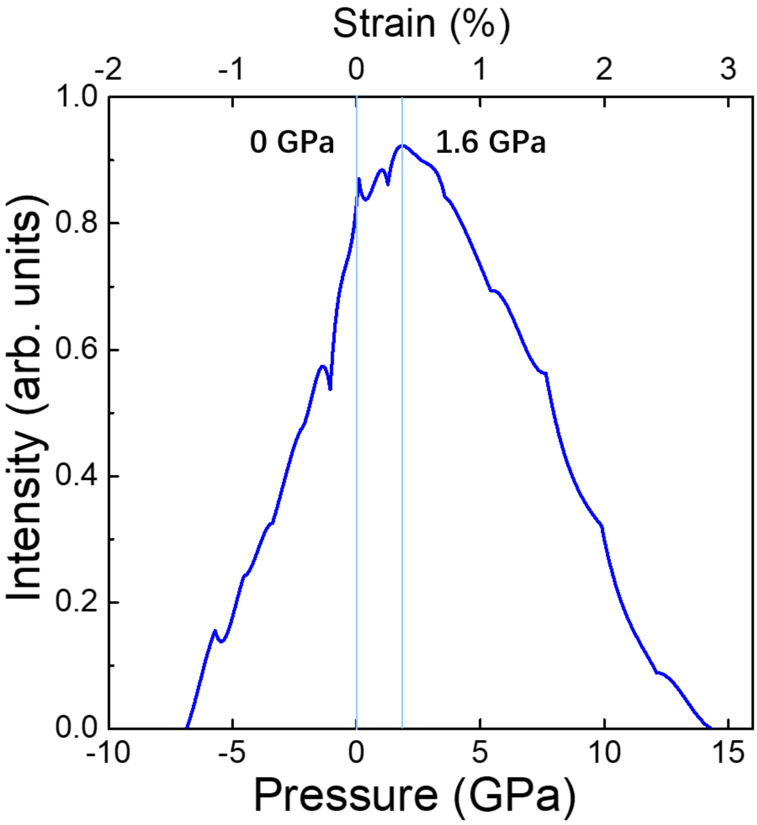

Supplementary Figure 4. The result of deconvolution of G peak of Raman spectrum between the initial CNT and the collapsed phase CNT.

For the CNT in the collapsed phase, the broad G peak comes from the strength change of the C-C bond, which attributes to the uneven external pressure. Considering the G peak position shift totally comes from the external pressure on the CNT, we can carry out the deconvolution Raman spectrum between the initial CNT and the collapsed phase CNT to estimate the pressure distribution in collapsed CNT, as the following equation,

$$Y(\omega) = \int K(P) \cdot y(P, \omega)\ \mathrm{d}P \quad (1)$$

Here, Y(ω) is the G peak Raman spectrum of the collapsed phase CNT. y (P, ω) is the initial CNT Raman spectrum and its peak position is linearly dependent on the external pressure P in two segments (G-band blueshift at a rate of 5.4 cm^{-1} GPa^{-1} below 4.0 GPa and a much-reduced rate of 2.8 cm^{-1} GPa^{-1} above 4.0 GPa). The pressure distribution K(P) has been shown in the Suppl. Fig. 4. The peak pressure is around 1.6 GPa and the

maximum pressure reach to ≈10 GPa, which is good agreement with the MD simulation results.

Suppl. Note 5. Few-layer graphene encapsulated CNT

Here, we replace hBN with few-layer graphene (FLG) and measure the height of FLG-encapsulated SWCNTs. The results are as follows. The thickness of graphene ranges from 1.0 nm to 2.0 nm. Suppl. Fig. 5a shows the height comparison between the CNT in and out of the graphene encapsulation. The height of FLG-encapsulated CNT drops to around 0.7 nm, which agrees well with the result of hBN-encapsulation measurement. Suppl. Fig. 5b shows the distribution histogram of the height of FLG-encapsulated CNT. Nearly 69% of the FLG-encapsulated CNTs collapsed. We suppose that the lower yield results from the thinner thickness of graphene compared with hBN flakes.

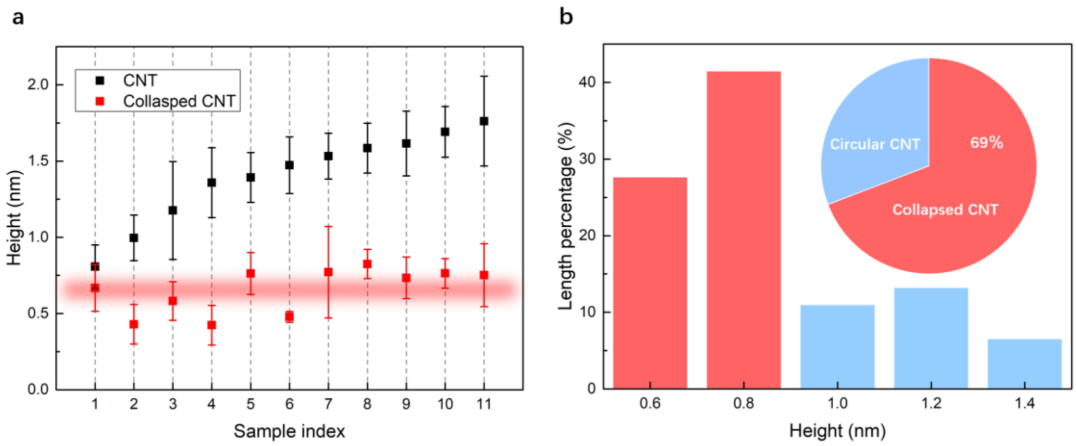

Supplementary Figure 5. The height statistics of FLG-encapsulated CNTs. a, the height comparison between the as-grown CNTs (black squares) and the FLG-encapsulated collapsed CNTs (red squares), where the error bar represents the standard deviation of multiple measurements of different positions. **b,** the distribution histogram of the height of FLG-encapsulated CNT on the SiO_2 substrate. The length percentage refers to the ratio between length of CNTs of specific height and the total length of all CNTs investigated.

Suppl. Note 6. Fully atomistic MD simulation of CNT encapsulated by hBN

The simulated model system consists of a 7.3 nm long (10,10) CNT, a large h-BN substrate, and a tri-layer top h-BN. During the simulation, the substrate is kept fixed.

The intra-layer interaction is computed via the AIREBO potential and Lennard-Jones potential for CNT, and Tersoff potential for h-BN. The interlayer interactions between the CNTs and the hBN layers are described via the registry-dependent ILP with refined parametrization.

Suppl. Fig. 6a shows the fully atomistic initial configurations of the encapsulated CNTs were generated via geometry optimization using the cg algorithm with a downward force (1 eV Å^{-1}) added on the edge of the tri-layer h-BN (dark blue atoms). The final configurations of the encapsulated CNTs were optimized with a threshold force value of 10^{-6} eV Å^{-1}. Suppl. Fig. 6b and c show the encapsulated results, front and side view, respectively. The simulation results of CNT encapsulated by h-BN simulated with about 30,000 atoms are similar to those of graphene encapsulate CNT in the main text.

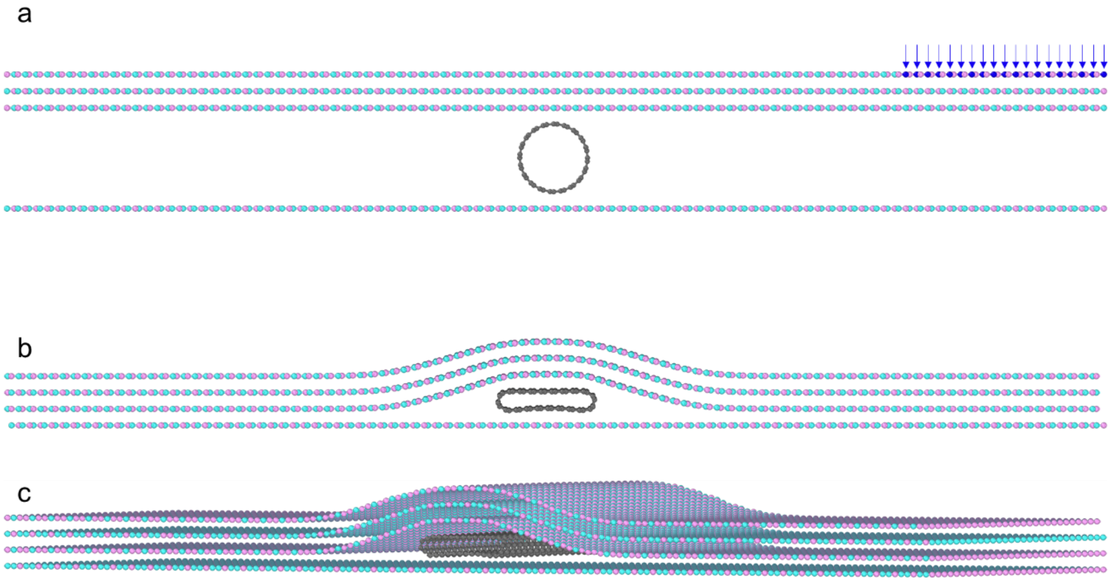

Supplementary Figure 6. The fully atomistic simulation of CNT encapsulated by h-BN. a, initial configurations, a downward force (1 eV Å^{-1}) is added on each dark blue atom. **b,** front view of the optimized structure of encapsulated CNT. c, side view of the optimized structure of encapsulated CNT. The gray dots represent the carbon atoms of the carbon nanotube, the blue ones represent the nitrogen atoms of the h-BN, and the red ones represent the boron atoms of the h-BN.

Suppl. Note 7. Theory of the Levy-Carrier (LC) model

In mechanics, the radial collapse pressure P_c for macroscopic tubes with a diameter of d_0, is known to scale as $P_c \propto d_0^{-3}$, as expressed by the Levy-Carrier (LC) theory. At

the nanoscale, it has been shown that such a formalism is consistent for CNT, when a correction term, $1-\beta^2/d_0^2$, is included. This term emerges both in simulations, as well as in experiments, and may be related to the large built-in curvature energy for small tube diameters, or to the discrete atomistic nature of tube walls. This approach is called the modified LC equation. With increasingly larger tube diameters, the correction term becomes less important, and the interaction of the external tube with the pressure-transmitting medium becomes more important. In order to account for the surrounding pressure transmitting medium (an argon bath in MD simulations), the energy per unit of tube length at the collapse pressure as the addition of the elastic energy has been introduced, the pressure term and the surface energy. As a result, the critical pressure can be described from the following equation

$$P_{\mathrm{c}}=\frac{24D}{d_0^3}\left(1-\frac{\beta^2}{d_0^2}\right)-\frac{2\gamma_{\mathrm{F-C}}}{d_0}, \qquad (2)$$

Where D (=1.7 eV) is the bending stiffness of graphene, $\gamma_{\mathrm{F-C}}$ (= 0.11 J m^{-2}) is the surface energy, and β (= 0.44 nm) corresponds to the diameter of the smallest free-standing stable CNT. Here, we can plot the phase diagram of CNT under pressure. Suppl. Fig. 5 shows the relationship between the diameter of CNT and its corresponding critical pressure calculated according to the modified LC equation. In the upper half of the curve, the pressure is bigger than the critical pressure $P>P_{\mathrm{c}}$, where the CNT is the collapse phase. When the surrounding pressure is zero, the minimum diameter of the collapse phase CNT is 5.1 nm, which is also the same as the experimental result. It is corresponding to the 8 nm width of double-layer GNR. When the vdW pressure is applied to the CNT, the minimum diameter of the collapse phase is reduced to 1.7 nm, which means under this VdW pressure most CNT can be compressed into the collapsed phase. The smallest diameter CNT in the collapsed phase is corresponding to the 2.7 nm width of double-layer GNR. Such narrow edge-closed GNRs experimentally result in higher FET performance.

Suppl. Note 8. Luttinger-liquid theory of plasmons in CNTs

We can compare the experiment results with the Luttinger liquid theory of plasmons. In Fig. 4f, the linear Luttinger liquid plasmon behavior can be observed, which shows the gate-independent plasmon behavior. For an individual suspended carbon nanotube of radius R screened by a concentric metal shell of radius R_S, the linear Luttinger liquid theory predicts the Luttinger liquid interaction parameter g to be $\frac{1}{g} = \frac{v_p}{v_F} = \sqrt{1 + \frac{8e^2}{4\pi\varepsilon_{eff}\pi\hbar v_F} \ln\left(\frac{R_S}{R}\right)}$, where v_p is the velocity of the collective charge mode, i.e., the plasmon velocity; v_F is the Fermi velocity; and ε_{eff} is the effective dielectric constant due to substrate screening. This parameter g is a function of Fermi velocity and the ratio R_s/R for a nanotube under a given dielectric environment. The plasmon wavelength λ_p for a given frequency f is related to g, as $\lambda_p=v_p/f=v_F/(gf)$. Because the Fermi velocity is a constant in metallic CNTs, all the Luttinger liquid phenomena related to g, including the plasmon excitations, will be independent of carrier density. As a result, the Luttinger plasmon wavelength in the metallic CNT is gate-independent, which matches well with our observation of the experiment results. The gate-tunable plasmon wavelength in semiconducting nanotubes from the hyperbolic band dispersion, where the Fermi velocity v_F depends on the carrier density. The Fermi velocity increases with higher Fermi energy. Due to the dominant strong repulsive interaction in CNTs, plasmon velocity v_p is approximately proportional to $\sqrt{v_F}$, and the plasmon wavelength in semiconducting nanotubes with band gap E_g depends on the Fermi wavevector k_F as follows:

$$\lambda_p = \lambda_{p0}\sqrt{\frac{v_F}{v_0}} = \lambda_{p0}\sqrt{\frac{\hbar v_0 k_F}{\sqrt{\left(E_g/2\right)^2+(\hbar v_0 k_F)^2}}} \quad (3)$$

where λ_{p0} and v_0 are the plasmon wavelength and Fermi velocity, respectively, in metallic nanotubes for a given frequency. In Fig. 4h, the experiment results show a good match with the fitting line from the nonlinear Luttinger liquid theory equation 2. In this fitting we have used $k_F = \frac{\pi}{4}n = \frac{\pi}{4}\kappa C_g|V_g|$, where n is the carrier density, C_g is the geometric capacitance and κ is a fitting parameter and indicates the overall gate efficiency. This result shows that the structural phase transition combined with metal-

semiconductor transition for the CNT.

Suppl. Note 9. Encapsulated CNTs simulated under different strains in the upper layer

We consider both tensile and compressive cases of the upper film separately by molecular dynamics simulations to account for some of the stresses that exist in real transfer processes. By increasing or decreasing the number of atoms in the upper film, we can modify the strain within the film.

In the case of tensile strain in the upper film, as shown in Suppl. Fig. 7, the shape of the carbon nanotube will hardly change because the van der Waals repulsion force between the layers is very large. The tension in the film will be uniformly distributed in the upper layer, the only difference is the envelope of the upper layer will vary depending on the strain.

In the case of compressive strain, as shown in Suppl. Fig. 8, the height of the encapsulated carbon nanotube gradually increases with increasing the number of atoms and is flattened laterally until finally the upper layer of the film tends to form a strain-free region and a wrinkle.

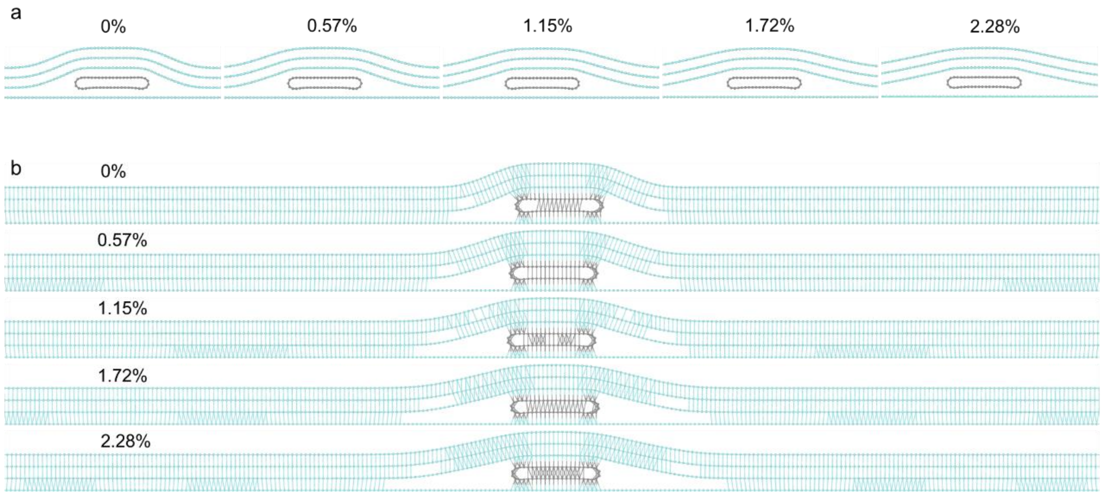

Supplementary Figure 7. The encapsulated carbon nanotube simulated under different tensile strains in upper layer. a, a zoom in view of carbon nanotubes. **b**, adds interlayer bonds (<0.35 nm) to assist in observing the relative positions of atoms between the lower film and the upper film. The gray dots represent the atoms of the carbon nanotube while the green ones represent the atoms of the upper film.

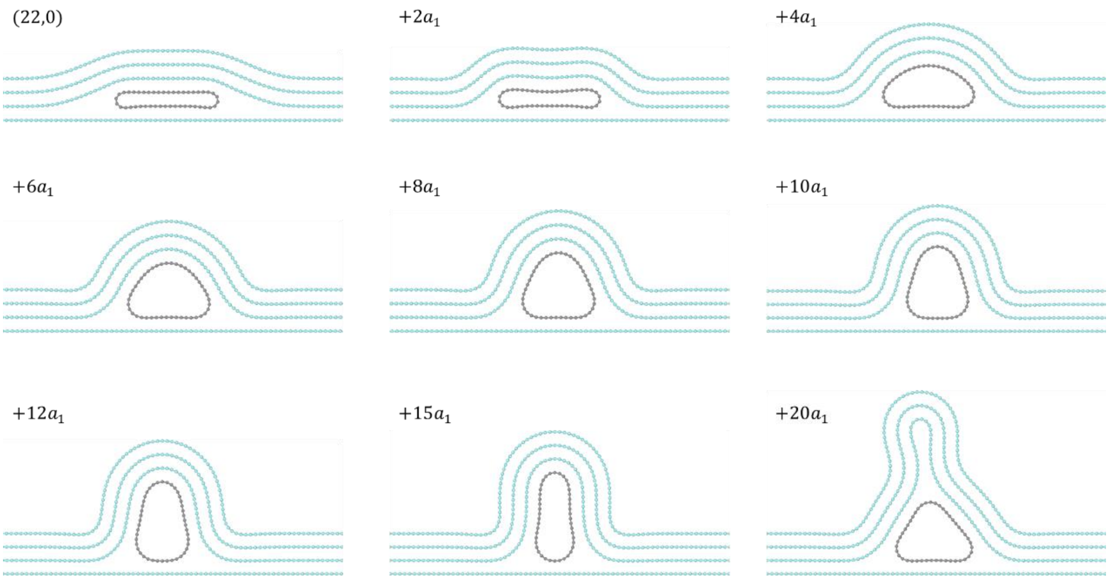

Supplementary Figure 8. Encapsulated carbon tubes simulated under different compressive strains in the upper layer. The gray dots represent the atoms of the carbon nanotube while the green ones represent the atoms of the upper film.

Suppl. Note 10. More examples for the collapse induced metal-semiconductor transition in CNTs

In the main text, we show only one example for the metallic to semiconducting transition in the structural collapse. In addition to the example presented in the main text, we have also observed the metallic to semiconducting transition in another 7 collapsed CNT samples, as displayed in Suppl. Fig. 9 below. In this study, we utilized scanning near-field optical microscopy (SNOM) to determine whether a CNT is metallic or semiconducting based on its response in the far infrared range. A metallic CNT exhibits a strong response and can support Luttinger-liquid plasmons in the far infrared range due to the existence of free charge carriers. Conversely, a semiconducting CNT has a much weaker infrared response. This method allows for the straightforward identification of a CNT's metallic or semiconducting nature. The yellow arrows in Suppl. Fig. 9 refer to the as-grown metallic CNTs, and the red arrows refer to the semiconducting part after collapse.

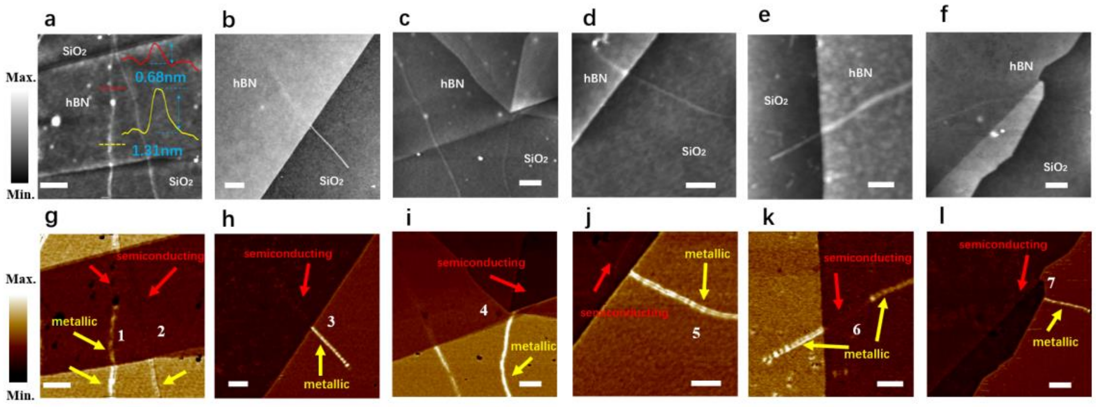

Supplementary Figure 9. Metallic CNTs that exhibits a semiconducting behavior after collapse. a-f, topography images of BN-encapsulated CNTs. The color bar represents the height. **g-l,** near-field optical images of the tubes shown in a-f. The yellow arrows refer to the as-grown metallic CNTs, and the red arrows refer to the semiconducting part after collapse. The color bar represents the intensity of near-field optical signal. Scale bars: 200 nm

Suppl. Note 11. Near-field optical map of typical distribution of hBN-encapsulated CNTs

Large-area images of CNTs are shown below in Suppl. Fig. 10. Red arrows refer to the hBN-encapsulated CNTs. From these images, one can see that the nanotube density is very low, typically a few micrometers apart from each other. The reason for such low tube density is the extremely low density of the catalytic Fe nanoparticles. As a result, the yielding nanotubes are mainly individual ones, and seldomly form bundles.

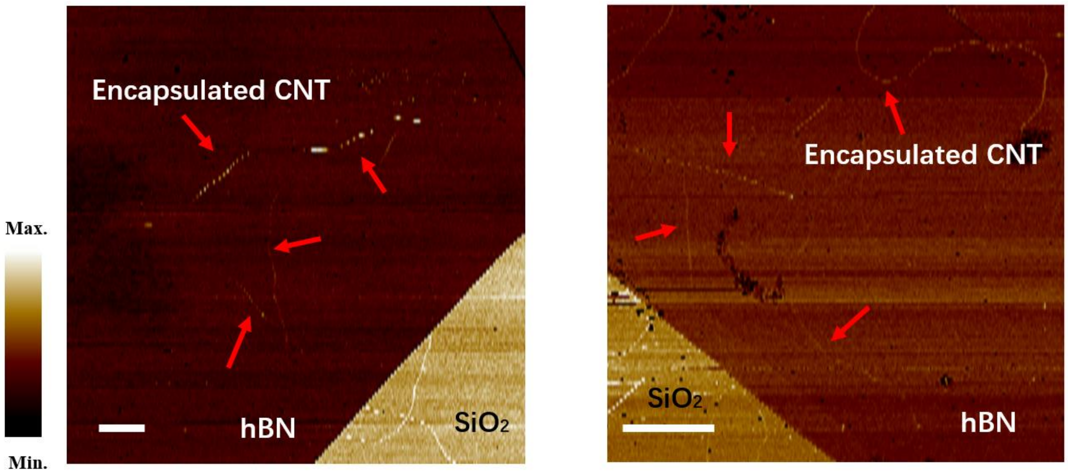

Supplementary Figure 10. The near field map of typical distribution of hBN-encapsulated CNTs. Red arrows refer to the hBN-encapsulated CNTs. The color bar represents the intensity of near-field optical response. Scale bars: 2 μm.
